# Adding to the debate on the numbers of options for MCQs: the case for not being limited to MCQs with three, four or five options

**DOI:** 10.1186/s12909-019-1801-x

**Published:** 2019-09-14

**Authors:** Mike Tweed

**Affiliations:** Department of Medicine, University of Otago Wellington, Wellington, New Zealand

**Keywords:** MCQ distractor

## Abstract

**Background:**

There is a significant body of literature that indicates that the number of options for single-best answer multiple choice questions (MCQs) can be reduced from five to three or four without adversely affecting the quality of the questions and tests. Three or four options equates to two or three distractors respectively.

**Maintext:**

Whilst these arguments may be true when focusing on psychometric aspects of questions, we should also focus on educational and clinical authenticity aspects of questions. I present reasons for MCQs in tests to have a variable number of options which will usually be more than three, four, or five. These include: decisions related to broad clinical scenarios cannot be limited to a small number of options; options lists should include all possible combinations of option elements; and options that are rarely chosen can provide information regarding students and/or for students.

**Conclusion:**

Finally, given computer based delivery, longer option lists are not impractical for examinees. In the contexts that are appropriate, it is time to consider a move to adopting appropriate and variable numbers of MCQ options and not be limited to MCQs with three, four or five options.

## Background

Multiple-choice questions (MCQs) are widely used in assessment within medical education and there are numerous articles comparing the number of response options and distractors [1]. Building further on this, it continues to be postulated that reducing the number of options does not lead to a reduction in assessment parameters [2, 3]. This creates a prevalent opinion as articulated in more recent reviews [4, 5] and primary research reporting that reducing the number of options does not result in significant differences in assessment parameters [6] or can lead to improvement in the parameters [7]. This body of literature suggests there are potential advantages and no disadvantages to reducing the number of MCQ options.

With this weight of evidence, why would assessment organisers not consider reducing to three or four options? In fact, to the contrary, I propose that assessment organisers consider having a variable number of options, which may mean increasing the number of options for many questions. The basis of this argument is that the evidence to reduce the number of options is based on a psychometric perspective, whereas the argument to have a variable number of options, which can include an increased number of options for many questions, is based on clinical authenticity and educational perspectives. In order to add to the debate, I proceed by way of presenting some reasons based on these perspectives.

## Main text

### Decisions related to broad clinical scenarios cannot be limited to a small number of options

Rarely do the questions faced by a clinicians in practice have exactly three, four or five options [8]. Although primarily developed because of concerns that a limited number of MCQ options would cue candidate to a correct response [8], longer lists of options are also perceived as being more authentic to clinical practice [9]. A single long list of options, hundreds of options long, could be used for all MCQs in an assessment [8, 10]. Equally, there is no reason the number of options has to be same for all questions in a test [11].

Extended matching type questions (EMQ) were developed such that without cueing from a short list of response options, clinical reasoning and knowledge may be assessed [12, 13]. An option list, of 5 to more than 25, is used for all questions, for a particular theme eg “What is the most likely diagnosis for a person presenting with chest pain?” [12]. An advantage of EMQs is that changes to the stem (patient scenario) can lead to a change in the correct answer from a longer list of options, thus reflecting clinical practice [14].

The number of options should not be defined by the format but the content of the question [15]. The number of options for a question should align with authentic clinical practice. There is not always the same number of options in clinical practice, so the number of response options should vary, and is likely to be more than three or four.

### Options lists should include all possible combinations of option elements

Some options for an MCQ might be made up of several descriptive elements. Rather than try to select which combinations of elements should be included or not, an alternative is to increase the number of options to ensure all combinations are included.

As an example, this is a question with eight possible combinations of elements:

“A person with breathlessness has the following blood gas analysis … ..”

Which option best describes the blood gas analysis?

A. Metabolic acidosis with a normal Aa (Alveolar-arterial) gradient
B. Metabolic acidosis with an increased Aa gradient
C. Metabolic alkalosis with a normal Aa gradient
D. Metabolic alkalosis with an increased Aa gradient
E. Respiratory acidosis with a normal Aa gradient
F. Respiratory acidosis with an increased Aa gradient
G. Respiratory alkalosis with a normal Aa gradient
H. Respiratory alkalosis with an increased Aa gradient

Rather than trying to select which three, four or five options should be included or not, it is possible to have all eight. As will be discussed subsequently, clinically important incorrect answers and psychometrically important incorrect answers might be different.

Where there are two elements with two possibilities, then there are four possible options to include [3]. This will also remove the futile hunt for a fifth option, when four options provides all plausible combinations of elements.

The number of options should include all combinations of elements, rather than limiting these to a set number of options for every question. The number of options will vary with the number of elements and therefore combinations, and is best supported by a policy of variable option numbers.

### Options that are rarely chosen can provide information regarding students and/or for students

Do we run the risk of losing important information if we remove rarely chosen options from MCQs? Many of the analyses upon which the recommendations to reduce the number of options are based on the assumption that incorrect responses do not have distinct intrinsic information. This is erroneous, there is significant information in incorrect responses, as there are responses that would be potentially unsafe if chosen in practice [16–21]. Panels of clinicians can consider the potential clinical impact of incorrect responses, which can lead to incorrect options being stratified for potential (un) safeness [16–21]. The most potentially unsafe responses are rarely selected [18, 20]. Rarely selected distractors are unlikely to be considered psychometrically important. Clinically important distractors are different from psychometrically important distractors [22]. Options that are rarely chosen can represent unsafe practices; it is vital to know which students are selecting these potentially unsafe responses [16–21]. Individual misconceptions can be included in feedback with the goal to direct personal learning development [16–21]. If they become apparent, cohort level misconceptions can be used with the goal to direct curriculum development. By removing rarely chosen but clinically important incorrect options representing potentially unsafe practices, we deny the opportunity for misinformed examinees to choose such options. The choice of unsafe options across multiple questions would be a concerning pattern that needs to be recognised to target learning and subsequent performance; should the pattern be repeated despite further learning opportunities, this information could be used to inform progression decisions [18, 20]. One postulated reason why examinees might continue to select unsafe options is the paucity of feedback they receive on answers that are unsafe as well as incorrect [18, 20].

The number of options in MCQs should be sufficient to include both psychometrically important distractors and clinically important distractors. As the number of each will not be the same for all content areas, their inclusion is likely to require more than three or four options, and is best supported by a policy of variable option numbers.

### Computer based delivery has made longer lists of options more feasible

Assessments do need to be practical and feasible [23]. Longer lists of response options might be difficult to fit on assessment documentation or for candidates to use. As already noted, MCQs with longer lists of options do not lead to impaired performance by examinees [8, 10]. A single long list of options, hundreds of options long, could be used, and such formats have proved feasible when facilitated by computer delivery [10], though it has also been implemented in a paper-based system [8].

As long as the question meets the cover test (the correct answer can be determined without seeing the options [24]), and the options are presented in a consistent logical order (e.g. alphabetical), then long lists are not a problem. Questions not meeting these standards are most likely to be flawed irrespective of the number of options.

With computer delivery of MCQ assessments, there is no space constraint on option lists, and each option is automatically set a corresponding response tick box. Computer marking mitigates errors in reading and grading responses.

## Conclusion

Now that many institutions are moving to computer delivery and marking of MCQ examinations, it is time to consider the move to adopting appropriate and variable numbers of MCQ options and not be artificially limited to MCQs with three, four or five options.

## Data Availability

Not applicable.

## Abbreviations

Aa: Alveolar-arterial
EMQ: Extended matching type questions
MCQ: Multiple choice question

## References

[1] Rodriguez MC (2005). Three options are optimal for multiple-choice items: a meta-analysis of 80 years of research. Educ Meas Issues Pract.

[2] Fozzard N, Pearson A, du Toit E, Naug H, Wen W, Peak IR (2018). Analysis of MCQ and distractor use in a large first year health Faculty Foundation program: assessing the effects of changing from five to four options. BMC Medical Education.

[3] Raymond MR, Stevens C, Bucak SD (2019). The optimal number of options for multiple-choice questions on high-stakes tests: application of a revised index for detecting nonfunctional distractors. Adv Health Sci Educ.

[4] Wilson I (2014). What's best for multiple-choice questions: three, four or five?. Clin Teach.

[5] Gierl MJ, Bulut O, Guo Q, Zhang X (2017). Developing, analyzing, and using distractors for multiple-choice tests in education: a comprehensive review. Rev Educ Res.

[6] Royal KD, Stockdale MR (2017). The impact of 3-option responses to multiple-choice questions on guessing strategies and cut score determinations. Journal of Advances in Medical Education & Professionalism.

[7] Kilgour JM, Tayyaba S (2016). An investigation into the optimal number of distractors in single-best answer exams. Adv Health Sci Educ.

[8] Veloski JJ, Rabinowitz HK, Robeson MR, Young PR (1999). Patients don't present with five choices: an alternative to multiple-choice tests in assessing physicians' competence. Acad Med.

[9] Huwendiek S, Reichert F, Duncker C, de Leng BA, van der Vleuten CP, Muijtjens AM, Bosse H-M, Haag M, Hoffmann GF, Tönshoff B (2017). Electronic assessment of clinical reasoning in clerkships: a mixed-methods comparison of long-menu key-feature problems with context-rich single best answer questions. Med Teach.

[10] Schuwirth L, Cvd V, Stoffers H, Peperkamp A (1996). Computerized long-menu questions as an alternative to open-ended questions in computerized assessment. Med Educ.

[11] Rogausch A, Hofer R, Krebs R (2010). Rarely selected distractors in high stakes medical multiple-choice examinations and their recognition by item authors: a simulation and survey. BMC Medical Education.

[12] Case SM, Swanson DB (1993). Extended-matching items: a practical alternative to free response questions. Teaching and Learning in Medicine: An International Journal.

[13] Beullens J, Struyf E, Van Damme B (2005). Do extended matching multiple-choice questions measure clinical reasoning?. Med Educ.

[14] Samuels A (2006). Extended matching questions and the Royal Australian and new Zealand College of Psychiatrists written examination: an overview. Australasian Psychiatry.

[15] Coderre SP, Harasym P, Mandin H, Fick G (2004). The impact of two multiple-choice question formats on the problem-solving strategies used by novices and experts. BMC Medical Education.

[16] Tweed M, Wilkinson T (2009). A randomized controlled trial comparing instructions regarding unsafe response options in a MCQ examination. Med Teach.

[17] Tweed MJ, Thompson-Fawcett M, Schwartz P, Wilkinson TJ (2012). A confidence and safety approach to MCQ scoring. Focus on Health Professional Education: A Multi-disciplinary Journal.

[18] Tweed M, Schwartz P, Thompson-Fawcett M, Wilkinson TJ (2013). Determining measures of insight and foresight from responses to multiple choice questions. Med Teach.

[19] Curtis DA, Lind SL, Boscardin CK, Dellinges M (2013). Does student confidence on multiple-choice question assessments provide useful information?. Med Educ.

[20] Tweed M, Stein S, Wilkinson T, Purdie G, Smith J (2017). Certainty and safe consequence responses provide additional information from multiple choice question assessments. BMC Medical Education.

[21] Rangel RH, Möller L, Sitter H, Stibane T, Strzelczyk A. Sure, or unsure? Measuring students’ confidence and the potential impact on patient safety in multiple-choice questions. Med Teach. 2017:1–6. 10.1080/0142159X.2017.1362103 28799435

[22] Swanson DB, Holtzman KZ, Allbee K (2008). Measurement characteristics of content-parallel single-best-answer and extended-matching questions in relation to number and source of options. Acad Med.

[23] Crossley J, Humphris G, Jolly B (2002). Assessing health professionals. Med Educ.

[24] Ware J, Vik T (2009). Quality assurance of item writing: during the introduction of multiple choice questions in medicine for high stakes examinations. Med Teach.

